# Intention Understanding in Human–Robot Interaction Based on Visual-NLP Semantics

**DOI:** 10.3389/fnbot.2020.610139

**Published:** 2021-02-02

**Authors:** Zhihao Li, Yishan Mu, Zhenglong Sun, Sifan Song, Jionglong Su, Jiaming Zhang

**Affiliations:** ^1^Institute of Robotics and Intelligent Manufacturing, The Chinese University of Hong Kong, Shenzhen, China; ^2^School of Statistics, Southwestern University of Finance and Economics, Chengdu, China; ^3^School of Science and Engineering, The Chinese University of Hong Kong, Shenzhen, China; ^4^Department of Mathematical Sciences, Xi'an Jiaotong-Liverpool University, Suzhou, China; ^5^School of AI and Advanced Computing, XJTLU Entrepreneur College (Taicang), Xi'an Jiaotong-Liverpool University, Suzhou, China; ^6^Research Center on Special Robots, Shenzhen Institute of Artificial Intelligence and Robotics for Society, Shenzhen, China

**Keywords:** human–robot interaction, intention estimation, scene understanding, visual-NLP, semantics

## Abstract

With the rapid development of robotic and AI technology in recent years, human–robot interaction has made great advancement, making practical social impact. Verbal commands are one of the most direct and frequently used means for human–robot interaction. Currently, such technology can enable robots to execute pre-defined tasks based on simple and direct and explicit language instructions, e.g., certain keywords must be used and detected. However, that is not the natural way for human to communicate. In this paper, we propose a novel task-based framework to enable the robot to comprehend human intentions using visual semantics information, such that the robot is able to satisfy human intentions based on natural language instructions (total three types, namely clear, vague, and feeling, are defined and tested). The proposed framework includes a language semantics module to extract the keywords despite the explicitly of the command instruction, a visual object recognition module to identify the objects in front of the robot, and a similarity computation algorithm to infer the intention based on the given task. The task is then translated into the commands for the robot accordingly. Experiments are performed and validated on a humanoid robot with a defined task: to pick the desired item out of multiple objects on the table, and hand over to one desired user out of multiple human participants. The results show that our algorithm can interact with different types of instructions, even with unseen sentence structures.

## 1. Introduction

In recent years, significant progress has been achieved in robotics in which human–computer interaction technology plays a pivotal role in providing optimal user experience, reduces tedious operations, and increases the degree of acceptance of the robot. Novel human–computer interaction techniques are required to further advance the development in robotics, with notably the most significant one being a more natural and flexible interaction method (Fang et al., 2018, 2019; Hatori et al., 2018). It requires robots to process external information as a human in many application scenarios. For home service robots, visual and auditory information is the most direct way for people to interact and communicate with them. With continual advancement in statistical modeling, speech recognition has been widely adopted in robots and smart devices (Reddy and Raj, 1976) to realize natural language-based human–computer interaction. Furthermore, substantial development in the field of image perception has been carried out, even achieving human-level performance in some tasks (Hou et al., 2020; Uzkent et al., 2020; Xie et al., 2020). By fusing visual and auditory information, robots are able to understand human natural language instructions and carry out required tasks.

There are several existing home service robots that assist humans in picking up specific objects based on natural language instructions. (Kollar et al., 2010) proposed to solve this problem by matching nouns and the target objects. Eppe et al. (2016) focuses on parsing natural language instructions by Embodied Construction Grammar (ECG) analyzer. Paul et al. (2018) utilizes probabilistic graph models for natural language comprehension, but objects are required to be described in advance through natural language. With the development of neural networks, some researchers tried to tackle the problem of natural language comprehension as a classification problem and connect the natural language representations of objects with objects in images (Matuszek et al., 2014; Alonso-Martín et al., 2015), although it turned out that classification plays an important role, and they rely on human intervention heavily, leading to less autonomous level. Shridhar et al. (2020) proposes an end-to-end INGRESS algorithm to generate textual descriptions of the objects in the image, and then relevancy clustering is performed with the object descriptions of human instructions for extracting the object with the highest matching score. Additionally, for multiple ambiguous objects, the robot can remove the ambiguity by identifying the objects. Hatori et al. (2018) uses the Convolutional Neural Network (CNN) and Long short-term memory (LSTM) to extract the features of the image and the text, respectively, and subsequently fuses visual and auditory information by a multi-layer perceptron. Magassouba et al. (2019) employed the Multimodal Target-source Classifier Model (MTCM) to predict region-wise likelihood of the target for selecting the object mentioned by instructions. Some works learn models for color, shape, object, haptics, and sound with predefined unique feature channels have resulted in successful groundings (Mooney, 2008; Dzifcak et al., 2009; Richards and Matuszek, 2019) explores using a set of general features to learn groundings outside of predefined feature channels. Despite these methods being relatively flexible to determine the target object described by natural language instructions, they cannot enable robots to understand the connections between different concepts. The capacity of understanding these connections determines the adaptability and flexibility of processing unstructured natural language instructions. If robots are able to flexibly parse and infer natural language sentences, users may have better experiences. For example, we expect robots to understand that “I am thirsty after running that far in such a hot day” means “Grasp a bottle to me,” and “I need to feed the little rabbit” means “Grasp a carrot to me.”

In order to achieve this goal, we propose a task-based framework combining both visual and auditory information to enable robots understand human intention from natural language dialogues. We first utilize the conditional random field (CRF) to extract task-related information from instructions, and complement a number of new sentences based on the matching rule. Then we apply Mask R-CNN (He et al., 2017) for instance segmentation and classification, and use sense2vec (Trask et al., 2015) to generate structured robot control language (RCL) (Matuszek et al., 2013); RCL is a robot-executable command for instruction. It represents the high-level execution intended by the person. It enables robots to perform actions in the specified tasks satisfying human requirements. To evaluate the efficacy of our approach, we classify human instructions into the following three types: Clear Natural Language Instructions, saying object names or synonyms clearly; Vague Natural Language Instructions, only providing object characteristics (hypernyms, related nouns, related verbs, etc.) without saying their names or synonyms; Feeling Natural Language Instructions, describing feelings of users in the scene without saying object names or synonyms. In such a manner, by transforming unstructured natural language instructions into robot-comprehensible structured language (RCL), robots can understand human intentions without the restriction of explicit expressions, and can comprehend connections between demand concepts and objects.

## 2. Methods

### 2.1. Image Recognition

In this work, we mainly use the Mask R-CNN for image recognition. The Mask R-CNN is improved on the basis of Fast R-CNN (Girshick, 2015) and Faster R-CNN (Ren et al., 2015). The architecture of Faster R-CNN integrates feature extraction, region proposal selection, bounding box regression, and classification, resulting in a significantly enhanced speed of object detection. The Mask R-CNN is inspired by Faster R-CNN with outputting both bounding boxes and binary masks, so object detection and instance segmentation are carried out simultaneously. In our work, we employ Resnet101-FPN as a backbone and use the result of instance segmentation as the image region to be matched, including the target object and the delivery place.

### 2.2. Information Extraction From Natural Language Instructions

We first use a rule matching method for preliminarily extracting natural language information. Furthermore, this method provides labels for the conditional random fields process to reduce labor intensity.

#### 2.2.1. Rule Matching

Rule matching uses linguistics as a fundamental principle to segment statements and label sentence components with predefined semantic information. The reason why rule matching is effective in parsing languages is that the languages are regular when they are restricted to a specific domain. Specifically, according to grammatical features, the sentence type is straightforward to identify, and the local feature of specific sentence types can be further utilized to extract key information. In this paper, two variables, i.e., lexical and dependency analysis, are selected. Compared to many existing studies with grasping robots, ours not only contain the single verb phrase-centered imperative sentence structure but also add many common sentence types for expressing intentions through natural language in the training set. These common sentences are selected from the three types described in section 1. The details of rule matching connecting sentence structure and instruction types are displayed in Table 1.

**Table 1:** Skills and details of the skills.

**Instruction type**	**Sentence structure**	**Target object**	**Delivery place**
Feeling type	Subject (user) + tether verb + epithet + other components	Words that are adjective and begin with a tethered verb in dependency analysis, words that are adjective and begin with an adverb in dependency analysis, etc.	Words that are personal pronouns and end in a nominal subject in dependency analysis, etc.
Vague type	Subject + modal verb + intransitive verb + other components	Words that are verbs and are the end of an open subordinate complement in dependency analysis, etc.	Words that are personal pronouns and end in a nominal subject in dependency analysis, etc.
Clear/Vague type	Subject + modal verb + transitive verb + noun + other components	Words that are common nouns and plural nouns, and are at the end of the direct object in dependency analysis.	Words that are personal pronouns and end in a nominal subject in dependency analysis, etc.
Vague type	Predicate + direct object (something) + indirect object + definite article (adjective or verb infinitive) + other constituents	Words that are verbs and end in a modifier in dependency analysis, words that are adjectives and end in an adjective modifier in dependency analysis, etc.	Words that are personal pronouns and end in a noun subject in dependency analysis, words that are personal pronouns and end in an indirect object in dependency analysis, etc.
Feeling type	It (for weather) + verb past tense or verb present progressive + other components	Words that are in the past tense of the verb and begin with the noun subject in dependency analysis, words that are in the present tense of the verb and begin with a non-primary verb in dependency analysis, etc.	System default users, etc.

#### 2.2.2. Conditional Random Fields

Although the rule matching method extracts key information from natural language with sufficient accuracy, it is inadequate because it still requires grammatical features to identify sentence types before parsing natural language. However, when the length and complexity of the instructions increase, the fixed rule may classify sentence types of the instructions incorrectly or extract unexpected information because of the interference by redundant information. Besides, high-frequency word features are not contained in the grammatical rule due to the limited and time-consuming enumeration work. Therefore, for further extraction of natural language information, a statistical model is necessary to integrate grammar and high-frequency words for mining specific local features.

We use the CRF model for information extraction, whose training data are labeled by the rule matching described previously. The process of extracting information from a sentence can be considered as sequence labeling. The model analyzes input natural language sequences, i.e., sentences, and outputs the label corresponding to each word. In this paper, the tag set is item, target, none, where “item” represents the keyword of the target object, “target” corresponds to the keyword of the delivery place, and “none” is the other components of the sentence.

The CRF is a common and efficient method for addressing the sequence labeling problem, and its principle is based on a probabilistic vectorless graph. In this paper, any sentence *x*(*x*1, *x*2, ......, *xn*) has 3*n* possible label sequences *y*(*y*1, *y*2, ......., *yn*), where (*xi, yi*) represents (word, word label). The probability of labeled sequence *y* is written as:

(1) $$\begin{array}{c} p(y|x)=\frac{e^{score(y|x)}}{\sum_{y^{\prime }}e^{score(y^{\prime }|x)}} \end{array}$$

(2) $$\begin{array}{l} score\left(y|x\right)=\overset{m}{\underset{j=1}{\sum }}\overset{n}{\underset{i=1}{\sum }}\lambda_{j}f_{j}\left(x,i,j\right) \end{array}$$

where *f*_*j*_(*x, i, j*) is *j*^*th*^ feature function at position *i* and usually is a binary function, generated by a feature template, which is broader in this study according to the variety of the instructions. At position *i*, (*y*|*x*) takes 1 when it satisfies the *j*^*th*^ feature function, otherwise takes 0. Parameter λ is the parameter to be learned. The objective of training model is to maximize the probability of the correctly labeled sequence. The size of m depends on the variety of training corpus, the number of variables, and the maximum offset.

### 2.3. RCL Generating

**Figure 1:**
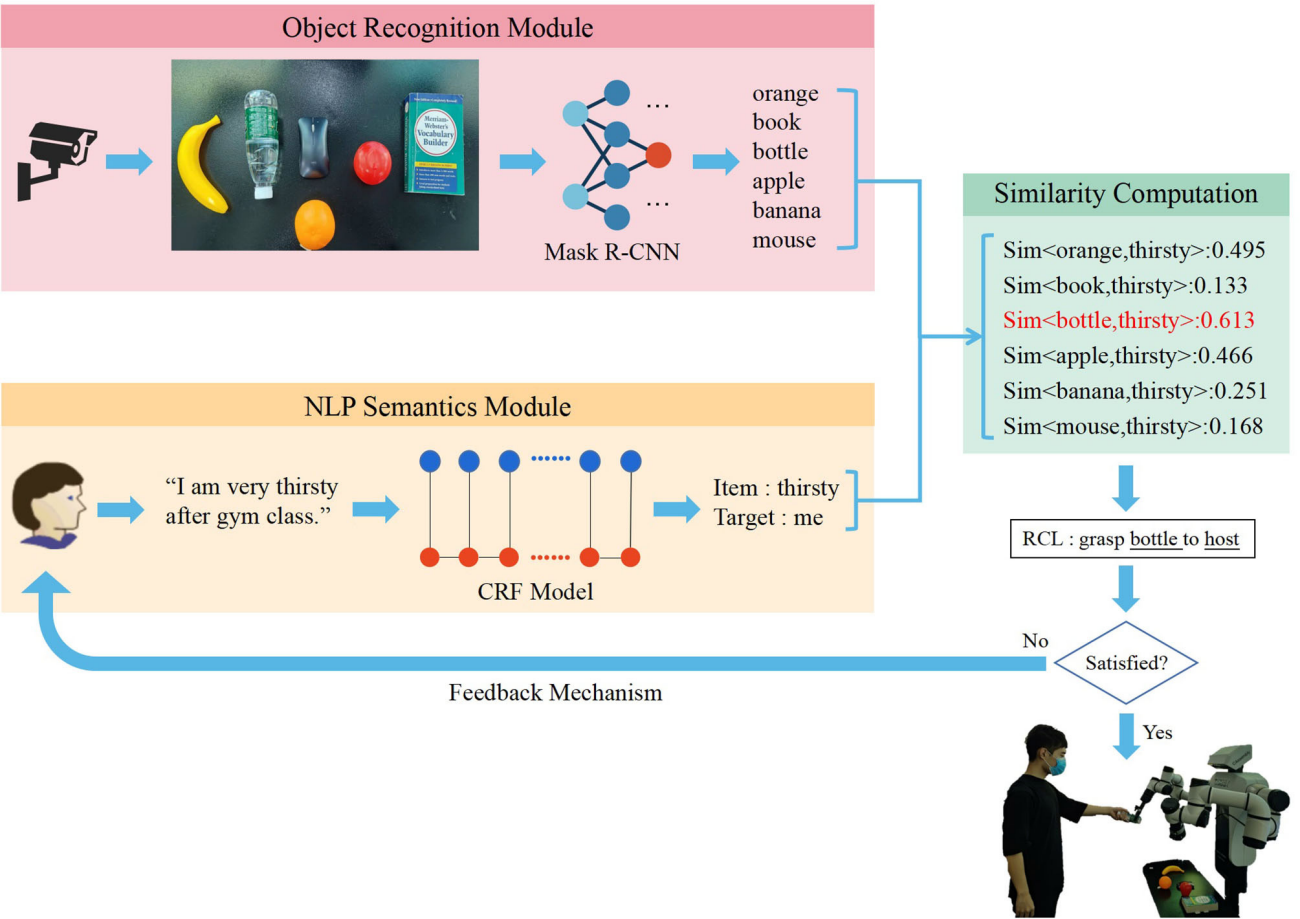
The overall framework: Once natural language information extraction and image recognition are completed, the features of natural language and the image are transformed into the same space for finding the object with the highest matching score. We transform both the object recognized from the image and the task-related information into word vectors to obtain the word vectors with maximum similarity. The vectors are then put into RCL (grasp object to place) to generate the structured language comprehended by robots.

In order to enable the robot to understand the highly arbitrary instructions provided by users and to grasp the target object to the delivery place, unstructured natural language instructions should be transformed into structured RCL. The RCL format utilized in this paper is “Grasp A to B,” where A and B represent the target object and the delivery place, respectively. In this work, the RCL format is generated from natural language instructions by extracting the keyword of the target object and place based on the information extraction module of CRF. Simultaneously, the image recognition module of Mask R-CNN is utilized for instance segmentation and classification. We map the extracted features of natural language instructions and images in the same feature space, and compare the degree of match between each object and two keywords. The two objects with the highest scores are A and B for generating the structured RCL language, “Grasp A to B.” The overall framework is shown in Figure 1.

We use the sense2vec model, which is an improved version of word2vec model, to transform the key information of images and natural language instructions to the same feature space. When words are fed into this model, the corresponding sense information is also required. Compared to the word vectors computed without context, those generated by sense2vec model contain contextual information and single vectors of corresponding compound words. Hence, the sense2vec model has more flexibility than the word2vec model. The sense2vec model employs CBOW, SG and structure-SG of word2vec, and uses token rather than a word as a semantic unit. Moreover, the same tokens with different tags are considered as different semantic units. The training process of the model is twofold. First, every token is labeled by a sense tag in the corresponding context. Second, the common models of word2vec, e.g., CBOW and SG, are fitted to the labeled data of the first step.

After the sense2vec model is used to obtain the objects according to the similarity between the information of target objects and object names in the scene, the degree of match is calculated. The object with the highest matching score is the target to grasp. We utilize cosine similarity, which is commonly used in word vector models, as an indicator of the degree of match between the objects and the keywords in instructions. The similarity is calculated as Equation (3), where *ITEM* denotes the item in the image and *A* denotes the word that is extracted by CRF, and *V*(*w*) is the sense vector of *w*.

(3) $$\begin{array}{l} sim\;\left(ITEM,A\right)=\frac{V\left(ITEM\right)\cdot V\left(A\right)}{\left||V\left(ITEM\right)||\times ||V\left(A\right)|\right|} \end{array}$$

### 2.4. Feedback Mechanisms

To make the robot grasp the item that humans want and be more robust, our system uses a feedback mechanism. When a user gives an instruction, the robot determines the target object and delivery place according to the instruction, and it asks the user whether the result is right.

We divide user feedback into three types. The first type is positive feedback, the user thinks robot's judgment is right. In this situation, the robot grasps the target object to the delivery place. The second type is negative feedback without any other valid information. In this situation, the robot chooses the object with the second largest matching score as the target object. The last type is negative feedback with other valid information. The algorithm uses CRF to extract the information related to the target object and uses sense2vec to calculate a new matching score between the information of target objects and object names in the scene, and then it chooses a new target object according to the updated matching score. The new target object is chosen by the following formula:

(4) $$object=\underset{i}{\arg\min}(\sum_{j=0}^{n}sim(item_{i},A_{j}))$$

where *object* denotes the target object, and *item*_*i*_ denotes the *i*^*th*^ item in the image, and *A*_*j*_ and *n* denote the word extracted by CRF in the *j*^*th*^ time and number of feedback, respectively, and *sim* denotes the similarity calculated by Equation (3).

For example, there is a scene with an apple, an orange, a banana, a bottle, and a book. The instruction is “I want to eat fruit.” Then the robot asks the user “Do you mean grasp the apple to host?” The feedback is “No, I want to eat something sour.” Algorithm can choose “sour” as valid information and use sense2vec to calculate a new matching score. Then it can grasp the orange to host.

### 2.5. Grasp Object

Current data-driven methods have significantly increased the accuracy of grasping objects (Mahler et al., 2016, 2019; Kalashnikov et al., 2018; Quillen et al., 2018) and they provide the technical basis for human–computer interaction.

We are inspired by a state-of-the-art method Dexnet4.0 (Mahler et al., 2019) and use end-effectors based on parallel gripper in the implementation of this study. We first generate a series of candidate grasps by pre-computation and utilize Grasp Quality Convolutional Neural Network (GQ-CNN) to score these grasps. The grasp with the highest score is implemented by robots. Since we only employ the parallel gripper, only pre-trained parallel gripper policy is utilized.

The full process of grasping is as follows. After the RCL is generated, the robot can use it to grasp the object. The RCL format in this paper is “Grasp A to B.” The system matches A and the results of image recognition. The matching result is a mask image. B is one of the predefined users. The mask image is the input of Dex-net2.0 that is used to determine the object to be grasped. Dex-net2.0 can generate a grasp position of the object. Then the robot arm will move to the position and grasp the object to the predefined user.

## 3. Results

We design experiments as follows. Microsoft COCO is a dataset for image recognition, and it provides many items that often appear in the home environment. We exclude items that are inappropriate to application scenarios from the Microsoft COCO (Lin et al., 2014). A total of 41 items remain and are categorized into 7 classes (animal, accessory, kitchen, sports, electronic, indoor, and food). Each experiment contains 3 categories of items and each category has some corresponding items, and we call it a scenario. Thus, there are altogether 35 scenarios, and each scenario includes more than 20 items. In each scenario, 8 subjects provide random instructions to the robots. Each subject provides 3 instructions containing the objects in the scene and lists of expected items for each instruction. There are 21 natural language instructions in each scenario, and 735 instructions in total. We show some examples of the collected instructions in Table 2.

**Table 2:** Examples of the collected instructions.

**Clear natural language instructions**	**Vague natural language instructions**	**Feeling natural language instructions**
Can I have a cup of tea?	I'm going to feed my monkey.	I am thirsty.
I want to play sports ball.	I need to control TV.	I am hungry.
I'm so thirsty that I need a large cup of cola.	The dark clouds shows that it will rain soon.	I'm tired.

### 3.1. Accuracy of Information Extraction

To enable robots to accurately parse complicated sentence structures, we apply the CRF model to extract information. The rule matching method is only for generating and evaluating the data of the CRF model. Therefore, quantitative evaluation of this method is not involved in this study.

We use 735 sentences collected before to test the accuracy of our CRF model's ability to extract the target object and the delivery place. We evaluate our CRF model in clear natural language instructions, vague natural language instructions, and feeling natural language instructions, respectively. The formula is as follows:

(5) $$\begin{array}{l} accuracy=\frac{\sum_{i=0}^{n}Is\text{\_}true\left(object_{i}\right)*Is\text{\_}true\left(place_{i}\right)}{n} \end{array}$$

where *accuracy* denotes the accuracy of the algorithm, and *Is*_*true* denotes whether the *object*_*i*_ is true. *n* denotes the number of instructions, and *object*_*i*_ and *place*_*i*_ denote the target object and place that are output by the algorithm.

The accuracy of the CRF model for clear natural language instructions, vague natural language instructions, and feeling natural language instructions are 0.710, 0.656, and 0.711, respectively. This result indicates that our method has consistent performance over all three types of instructions. By analyzing the failure cases, we found that the wrong inferred item and the wrong inferred target are most likely due to the deficiency in training data that reflect their local features. The local features are referred to words, positions, and dependency.

### 3.2. Evaluation of Human–Robot Interaction

**Figure 2:**
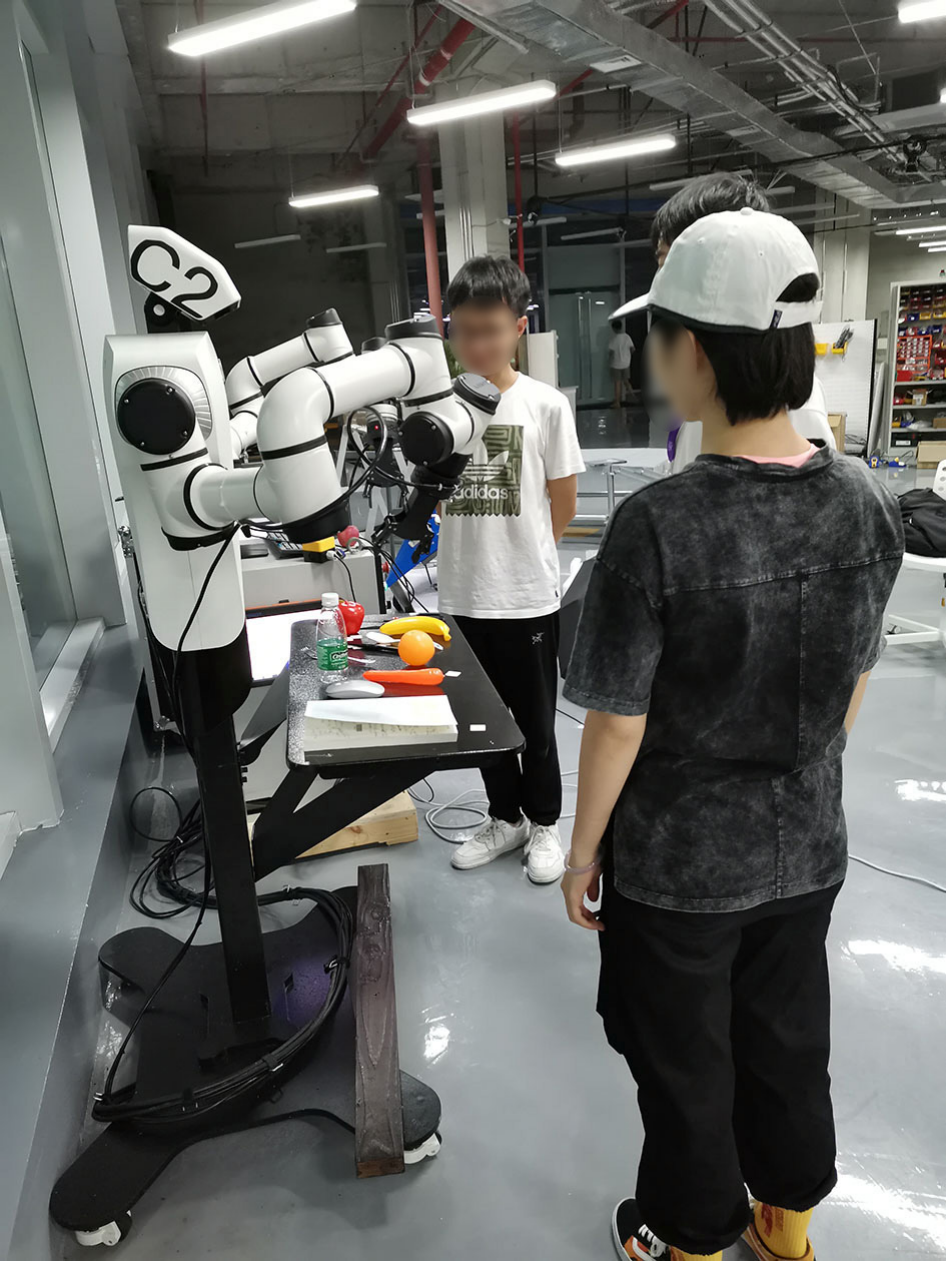
Experimental setup for robot experiments. Our system uses Cobot CAssemblyC2 for experiment.

To obtain meaningful results, we evaluate our system's human–robot interaction ability in the scenarios. There are 21 instructions that are provided by 8 subjects in each scenario. The experimental setup is shown in Figure 2.

Our system uses a feedback mechanism. The robot has a ranking list according to matching score. If a user gives negative feedback without any other valid information, the robot is able to choose the object with the second largest matching score as the target object, and so on. Therefore, we use reciprocal rank (RR) as the evaluation of our system. RR is a measure to evaluate systems that return a ranked list of answers to queries, and mean reciprocal rank (MRR) is the mean of the sum of RR. The formulas are given by:

(6) $$\begin{array}{l} RR_{i}=\frac{1}{Position\left(item\right)} \end{array}$$

(7) $$\begin{array}{l} MRR=\frac{\sum_{i=1}^{N}RR_{i}}{N} \end{array}$$

where *Position*(*ITEMi*) represents the position of the real target object in the matching score list, and *N* is the number of instructions in each scenario, and *RR*_*i*_ is the reciprocal rank of *i*th instruction within each scenario.

**Figure 3:**
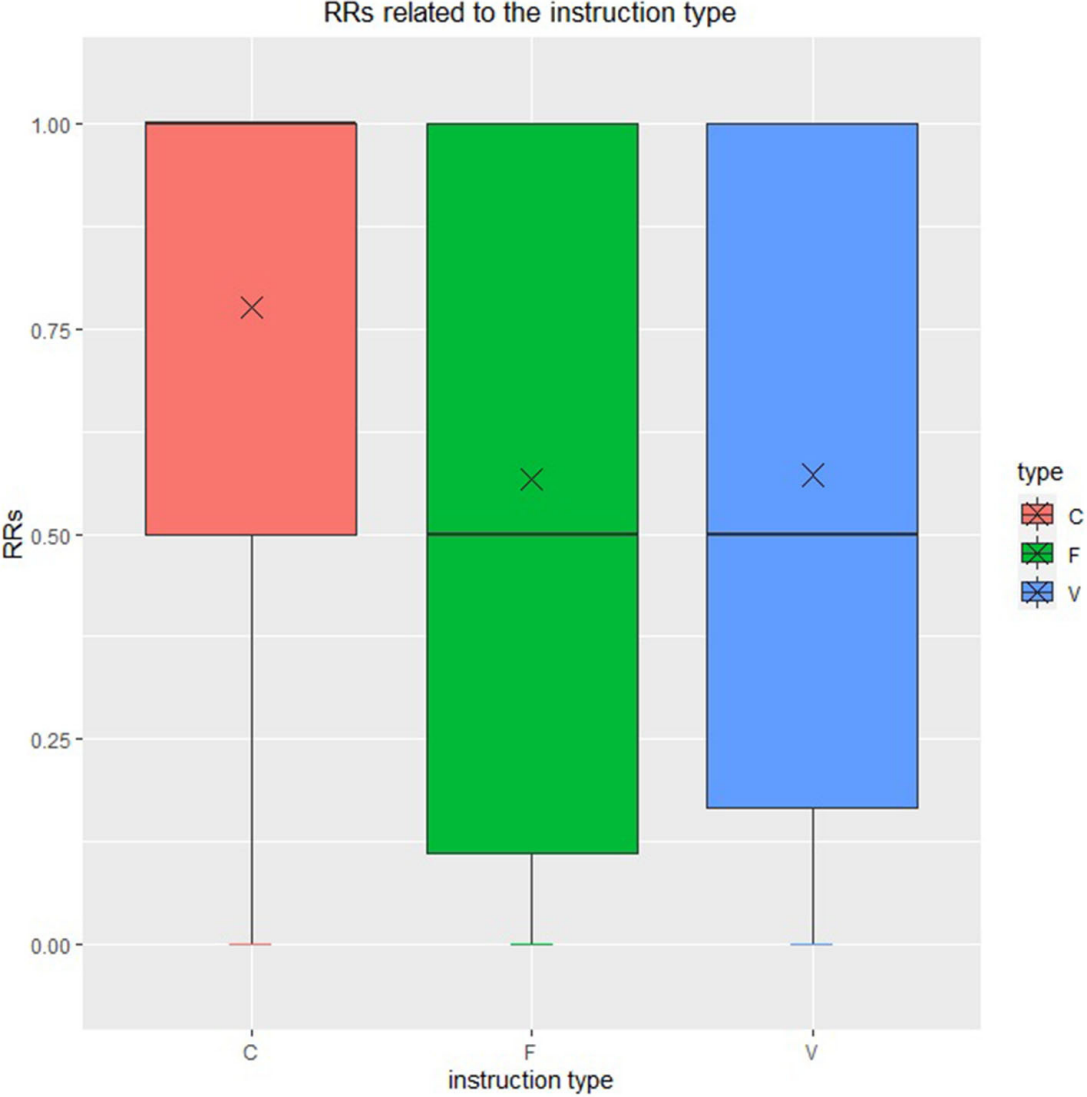
Type-specific reciprocal rank. Red, green, and blue represent clear natural language instruction, feeling natural language instruction and vague natural language instruction.

The distributions of type-specific RR are demonstrated in Figure 3. The mean reciprocal ranks of clear natural language instruction, feeling natural language, and vague natural language is 0.776, 0.567, and 0.572, respectively. The medians is 1 for clear natural language instruction, which shows that the robot can grasp the correct object at the first attempt according to clear natural language instruction in most cases. The mean reciprocal rank of all instructions is 0.617, which means the robot need about 1–2 attempts to grasp the correct object according to the three types of instruction at the average level. Thus, we draw a conclusion that the robots infer the expected item effectively, and especially, the robots make inference most effectively and most steadily according to clear natural language instructions among the three types of instructions defined as before. The result also shows our framework's ability to interact with people.

**Figure 4:**
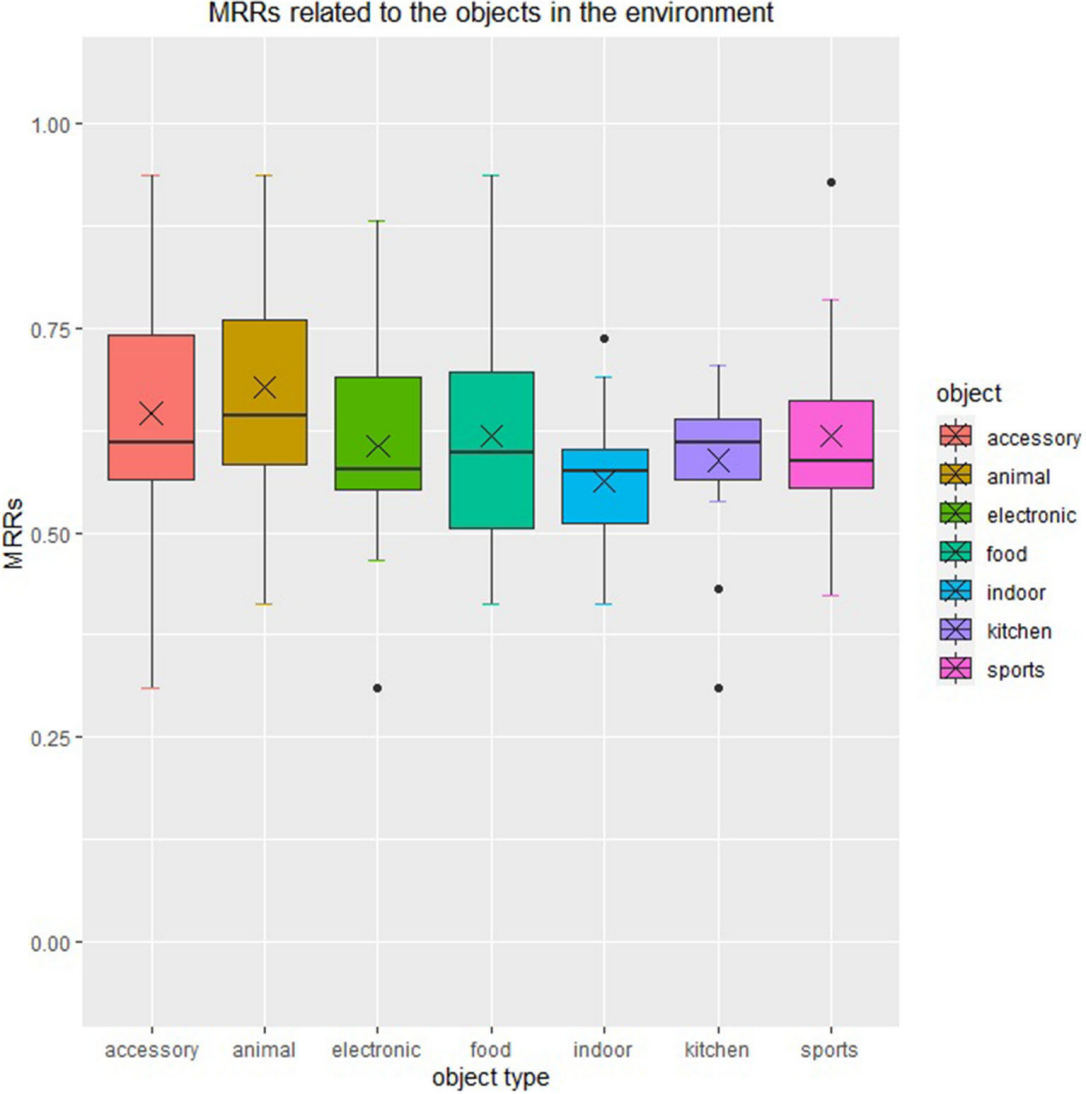
Scene-specific mean reciprocal rank. This figure shows the relevance between scene-specific reciprocal rank and the categories in each scene.

We group the MRR by categories in their corresponding scene, with intersections existing among groups. The result of our experiment is shown in Figure 4, which indicates that the robots perform best and relatively steadily when items in “animal” category appear in the scene, and perform worst and relatively unsteadily when items in “indoor” and “food” categories appear in the scene. It is because that the items in these categories always appear in a similar context. It is also related to the word embedding model.

**Figure 5:**
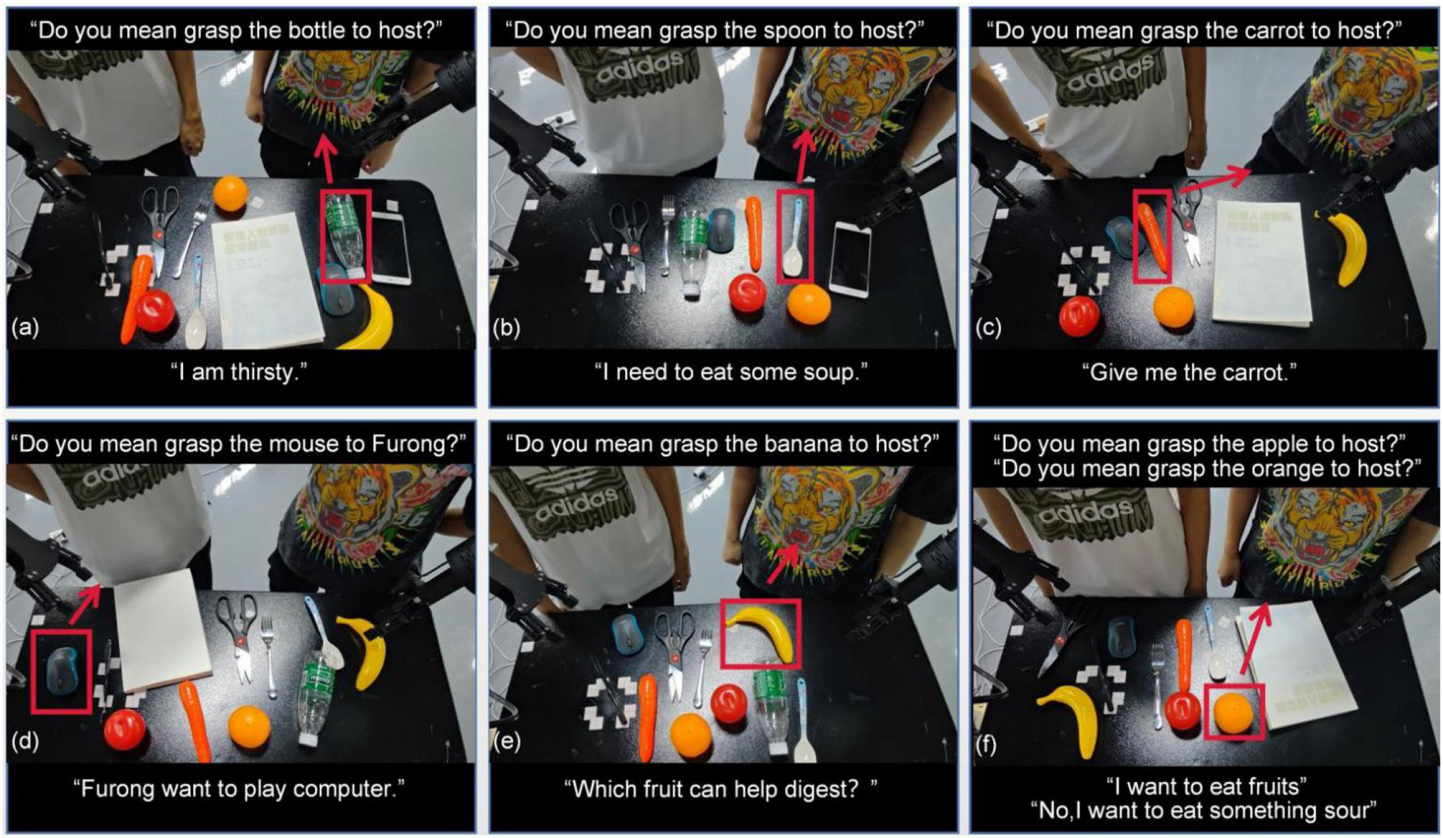
Some examples of human–robot interaction. There are two user in these scenarios. The one on the right is host, and the other one is Furong. Red boxes indicate the target objects chosen by our method. Red arrows indicate the delivery directions.

The human–robot interaction ability of our system is shown in Figure 5. Figures 5a–c illustrate the interaction for feeling natural language instruction, vague natural language instruction, and clear natural language instruction, respectively. Figure 5d illustrates that our method can grasp objects to a different user. Figure 5e illustrates our method's ability to adapt to instructions that have untrained sentence structures, which is an interrogative question in this case. Figure 5f shows the feedback mechanism of our method. The robot can grasp the orange because of the feedback information that says he wants to eat something sour.

### 3.3. The Ability to Deal With Unseen Sentence

We also note that this algorithm has a generalization capability to some extent. It can analyze a question like “Which item can help me use computers more efficiently?,” even though this sentence type is not involved in the training set. Therefore, we choose 104 instructions that have unseen sentence structures to test the generalization capability of our approach, such as interrogative sentences and complex sentences.

The mean reciprocal rank for instructions that have untrained sentence structures is 0.483, which means the site of the target object is in the second position in the recommended list on average, and the robot can grasp the correct object with about 2–3 attempts at the average level.

This also shows that our model has a generalization capability to interact with complex instructions that have unseen sentence structures.

## 4. Conclusion

Our proposed algorithm transforms unstructured natural language information and environmental information into structured robot control language, which enables robots to grasp objects following the actual intentions of vague, feeling, and clear type instructions. We evaluate the algorithm performance using a human–robot interaction task. The experimental results demonstrate the ability of our algorithm interacting with different types' instructions and a generalization ability of unseen sentence structures. Although some sentence types are not involved in the training set, the carried information still can be effectively extracted, leading to reasonable intention understanding.

In our future work, we would construct the databases based on multiple tasks to extend its skill coverage, and explore its potential in understanding more complex tasks.

## Data Availability Statement

The raw data supporting the conclusions of this article will be made available by the authors, without undue reservation.

## Ethics Statement

Ethical review and approval was not required for the study on human participants in accordance with the local legislation and institutional requirements. The patients/participants provided their written informed consent to participate in this study.

## Author Contributions

ZL proposed the framework to enable the robot to comprehend human intentions from vague natural language instructions. ZS, ZL, and YM modified and finalized the design of this framework. ZS also proposed the feedback mechanism that improve the robust of our algorithm. YM and ZL collect the data of instructions, and they also conducted the experiment together. SS and JS helped edit this paper, and they give us many useful advice. JZ provided theoretical guidance for the proposed method and experiments design. All authors contributed to the article and approved the submitted version.

## Conflict of Interest

The authors declare that the research was conducted in the absence of any commercial or financial relationships that could be construed as a potential conflict of interest.
